# Bone Turnover Marker (BTM) Changes after Denosumab in Giant Cell Tumors of Bone (GCTB): A Phase II Trial Correlative Study

**DOI:** 10.3390/cancers14122863

**Published:** 2022-06-10

**Authors:** Emanuela Palmerini, Laura Pazzaglia, Luca Cevolani, Loredana Pratelli, Michela Pierini, Irene Quattrini, Elisa Carretta, Maria Cristina Manara, Michela Pasello, Giorgio Frega, Anna Paioli, Alessandra Longhi, Marilena Cesari, Rossella Hakim, Toni Ibrahim, Laura Campanacci, Eric Lodewijk Staals, Davide Maria Donati, Maria Serena Benassi, Katia Scotlandi, Stefano Ferrari

**Affiliations:** 1 Osteoncology, Bone and Soft Tissue Sarcoma and Innovative Therapy, IRCCS Istituto Ortopedico Rizzoli, Via Pupilli 1, 40136 Bologna, Italy; michela.pierini@ior.it (M.P.); elisa.carretta@ior.it (E.C.); giorgio.frega@ior.it (G.F.); anna.paioli@ior.it (A.P.); alessandra.longhi@ior.it (A.L.); marilena.cesari@ior.it (M.C.); rossella.hakim@ior.it (R.H.); toni.ibrahim@ior.it (T.I.); stefanoferrari.19855@gmail.com (S.F.); 2 SSD Laboratory of Experimental Oncology, IRCCS Istituto Ortopedico Rizzoli, Via di Barbiano 1/10, 40136 Bologna, Italy; laura.pazzaglia@ior.it (L.P.); mariacristina.manara@ior.it (M.C.M.); michela.pasello@ior.it (M.P.); mariaserena.benassi@gmail.com (M.S.B.); katia.scotlandi@ior.it (K.S.); 3 Third Orthopaedic Clinic and Traumatology, IRCCS Istituto Ortopedico Rizzoli, Via Pupilli 1, 40136 Bologna, Italy; luca.cevolani@ior.it (L.C.); laura.campanacci@ior.it (L.C.); ericlodewijk.staals@ior.it (E.L.S.); davide.donati@ior.it (D.M.D.); 4 Department of Pathology, IRCCS Istituto Ortopedico Rizzoli, Via Pupilli 1, 40136 Bologna, Italy; danapratelli@gmail.com; 5 Scientific Direction IRCCS Istituto Ortopedico Rizzoli, Via Pupilli 1, 40136 Bologna, Italy; irene.quattrini@ior.it

**Keywords:** giant cell tumor of bone, denosumab, bone turnover markers, carboxyterminal crosslinked telopeptide of type I collagen

## Abstract

**Simple Summary:**

Giant cell tumors of bone (GCTB) are a histologically benign, yet often aggressive, skeletal tumor. Surgery is the only potentially curative treatment option, but may be associated with severe morbidity and loss of function. Denosumab, a monoclonal antibody directed to RANK ligand, is approved for GCTB treatment. Here we report the results of a phase II correlative study on sBTMs (carboxytermnal-crosslinked-telopeptide of type I collagen (s-CTX), alkaline phosphatase (ALP), bone-alkaline phosphatase (bALP), parathyroid hormone (sPTH), and osteocalcin (OCN) in GCTB patients treated with denosumab. The main finding of the present research is that the use of denosumab in patients with GCTB induces the bALP/ALP/OCN and s-CTX reduction, with a concomitant increase in sPTH. High baseline s-CTX identifies a group of patients at higher risk of progression of the disease. Stratification of the GCTB patients based on s-CTX might guide type of surgery and follow-up.

**Abstract:**

Background: Giant cell tumors of bone (GCTB) are osteolytic tumors. Denosumab, a RANK-L inhibitor, is approved for GCTB. Data on serum bone turnover marker (sBTM) changes are lacking. We present a phase II correlative study on sBTMs in GCTB patients treated with denosumab. Methods: All GCTB patients receiving denosumab within a multicentre, open-label, phase 2 study were enrolled. Serum levels of carboxyterminal-crosslinked-telopeptide of type I collagen (s-CTX), alkaline phosphatase (ALP), bone-alkaline phosphatase (bALP), parathyroid hormone (sPTH), and osteocalcin (OCN) were prospectively assessed (baseline, T0, 3 months, T1, 6 months, T2). The primary endpoint was assessment of sBTM changes after denosumab; the secondary endpoints were disease-free survival (DFS) and sBTM correlation. Results: In 54 cases, sBTMs decreased during denosumab treatment except for sPTH. With a median follow-up of 59 months, 3-year DFS was 65% (%CI 52–79), with a significantly worse outcome for patients with high (≥500 UI/mL) s-CTX at baseline, as compared to low s-CTX (<500 UI/mL) (3-year DFS for high CTX 45% (95%CI 23–67) vs. 75% (95%CI 59–91) for low s-CTX. Higher median ALP and s-CTX were found for patients with tumor size ≥ 5 cm (*p* = 0.0512; *p* = 0.0589). Conclusion: Denosumab induces ALP/OCN and s-CTX reduction. High baseline s-CTX identifies a group of patients at higher risk of progression of the disease.

## 1. Introduction

Giant-cell tumor of the bone (GCTB) is a benign but aggressive skeletal neoplasm, with a high recurrence rate of between 33 and 50% after curettage and resection [1,2]. The typical histological appearance is that of abundant giant cells with a benign spindle cell background [3]. Giant cells are osteoclast-like cells originating from hematopoietic stem cells and expressing tartrate-resistant acid phosphatase (TRAP), cathepsin K, calcitonin receptor and vitronectin receptor [4]. Cytogenetically, the most common chromosome aberrations are telomeric associations involving multiple chromosomes [5]. Recently, mutations of the H3F3A gene at the Gly34 codon, mostly G34W resulting from a GGG > TGG nucleotide change, have been identified in the vast majority of this tumor [6].

The standard treatment is curettage or wide resection. Preoperative arterial embolization, intralesional curettage, or radiation therapy are alternative procedures when curettage or resection are not feasible [7,8]. Lung metastasis from a GCTB is rare; it is observed in 2–10% of patients with GCTB [1]. Since 2013, a medical treatment with denosumab, Xgeva^®^, was approved for patients with GCTB.

Denosumab is a fully human monoclonal antibody against the receptor activator of nuclear factor κB (RANK) ligand (RANKL). RANKL facilitates the differentiation of osteoclast precursors into osteoclasts, resulting in bone resorption [9]. This is important in the regulation of bone growth and turnover. Several serum markers are available to assess the bone turnover [10]. The main serum bone turnover markers (sBTMs), can be classified in two groups: markers of bone formation, products of osteoblast activity bone-alkaline phosphatase (bALP) and osteocalcin (OCN), and markers of bone resorption, such as carboxyterminal-crosslinked-telopeptide of type I collagen (s-CTX) [11,12]. A key molecule in bone turnover is parathyroid hormone (PTH), a major endocrine inducer of bone remodeling that stimulates osteoblasts and indirectly activates osteoclasts by regulating receptor activator of NF-κB ligand (RANKL) and osteoprotegerin in osteoblast-lineage cells [13,14]. sBTMs showed good prognostic ability to predict bone-specific recurrence in breast cancer [15]. They also provided valuable prognostic information in patients with bone metastases receiving bisphosphonate [15].

Prospectively collected data on serum bone turnover marker (sBTM) changes after denosumab in patients with GCTBs are lacking. We present a phase II correlative study, conducted in one referral center. The primary endpoint was assessment of sBTM changes after denosumab; the secondary endpoints were disease-free survival (DFS) and sBTM correlation.

## 2. Materials and Methods

### 2.1. Design and Patients

All GCTB patients underwent imaging studies for diagnostic purposes including plain radiographs and MRI-scans. Histologic confirmation was obtained in all cases through a trocar needle biopsy, either under image intensifier or CT-guided. Prior to starting, and during, denosumab treatment, serial CT-scans of the bone lesion were performed every 3 months. Cases were discussed during a multidisciplinary tumor board for sarcomas, during which the treatment approach was determined.

All GCTB patients receiving denosumab (120 mg on days 1, 8, 15, 29, and every 4-weeks thereafter) within a multicenter, open-label, phase 2 study (NCT00680992) were enrolled.

Inclusion criteria for the phase II study were as follows: adults or skeletally mature adolescents (aged ≥12 years) weighing at least 45 kg with pathologically confirmed GCTB, a Karnofsky performance status of 50% or higher (or Eastern Cooperative Oncology Group status 0, 1, or 2), and measurable active disease within 1 year of study enrolment. Active disease was defined as imaging evidence of osteolysis within the tumor or increasing symptoms such as pain, in conjunction with histopathology showing evidence of giant cells. Patients with a known or suspected diagnosis of sarcoma, non-GCTB giant-cell rich tumors, brown cell tumor of bone, or Paget disease, history or current evidence of osteonecrosis or osteomyelitis of the jaw, or those who required oral surgery or had unhealed dental or oral surgery were excluded. Exclusion criteria also included the current use of alternative GCTB treatments (e.g., radiation, chemotherapy, embolization, or bisphosphonates). All patients provided written informed consent.

According to the protocol, patients were divided into two cohorts on the basis of surgical salvageability (cohort 1: unresectable, and cohort 2: resectable) (Table 1). Surgery after denosumab was scheduled after 6 months on treatment. Five patients judged resectable at study entry did not eventually undergo surgery and were continued on denosumab. The inclusion criterium for the present correlative sBTM study was the presence of bone disease; patients with lung-only disease were excluded.

Serum levels of s-CTX, ALP, bALP, sPTH, and OCN were prospectively assessed. Serum samples were taken at baseline and repeated at three-month intervals: baseline, T0, 3-months T1, and 6-months T2.

### 2.2. sBTM Expression Levels in Serum Analysis

Blood samples collected at different time points were centrifuged at 5000× *g* for five minutes to obtain serum samples. s-CTX, bALP and OCN levels were measured with enzyme linked immunosorbent assay (ELISA), ALP was measured with the colorimetric method, and the measurement of PTH was made by the chemiluminescence method. In particular, s-CTX was measured using β Crosslaps/serum kit (Roche Pharma AG, Reinacach BL, Swiss, Basel, Switzerland), ALP was measured using Alkaline Phospahte kit (Roche Pharma AG, Reinacach BL, Swiss); bALP was measured using Tandem–R Ostease kit (Becman Coulter Italia, Roma, Italy); sPTH was measured using PTH kit (Roche Pharma AG, Reinacach BL, Swiss) and OCN was measured by N-MID Osteocalcin kit (Roche Pharma AG, Reinacach BL, Swiss). All the analyses were performed by using Elecsys and Cobas 6000 instruments (Roche, Basel, Switzerland).

### 2.3. Statistics

Descriptive statistics were performed for all variables by median value and range, frequency and percentage. BMT analysis was conducted in the whole series (cohort 1 and 2). Baseline sBTM values were compared between tumor size (≤5 cm, >5 cm) and age (<50 years, ≥50 years) using the Wilcoxon–Mann Whitney test. Nonparametric repeated-measures analysis of variance (ANOVA), followed by the Tukey multiple comparison test at each time point were used to evaluate sBTM expression level over time.

Threshold values were set to the sBTM median value and patients were stratified into two groups (high and low expression).

Kaplan–Meier analysis on DFS was performed; an event was defined as recurrence of disease after surgery. For all tests, *p* < 0.05 was considered significant. All statistical analyses were performed with IBM SPSS Statistic 21.0 (IBM™ Corp, Armonk, NY, USA).

## 3. Results

### 3.1. Clinical Presentation

**Figure 1:**
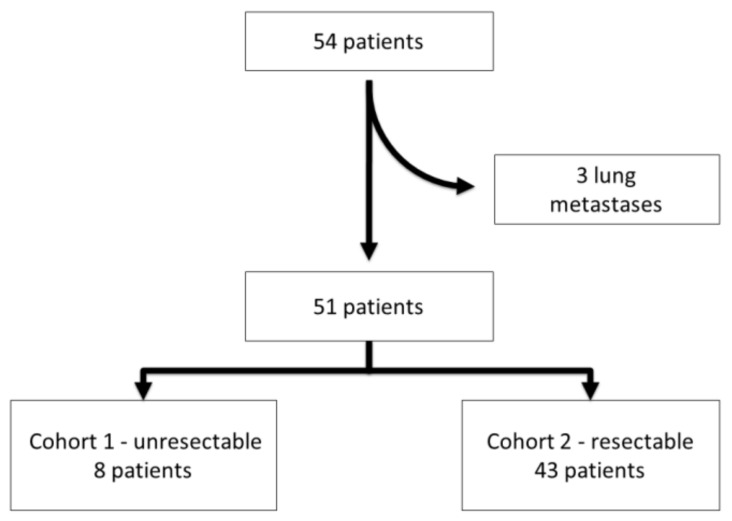
The flow chart of patient selection process.

**Figure 2:**
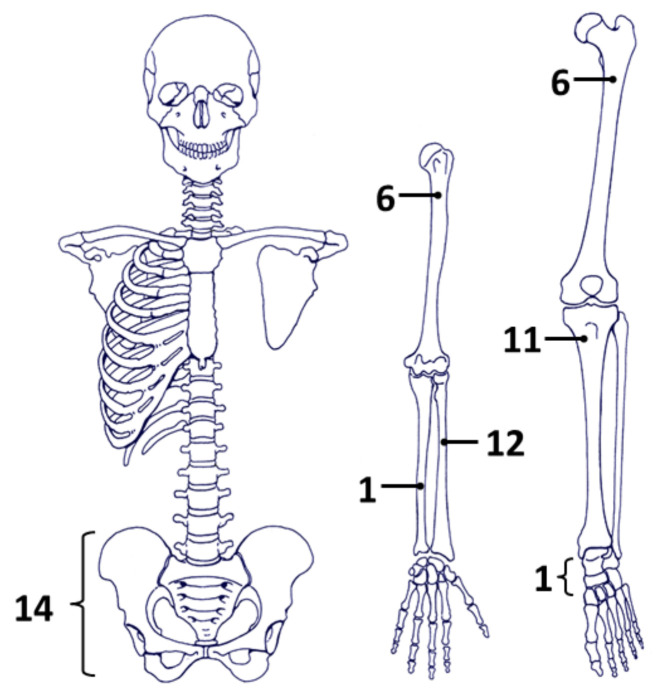
Anatomical site distribution of the 51 GCTB in the 51 patients enrolled.

From 2006 to 2015, 54 cases were identified. Three cases with lung metastases and no bone involvement at the time of treatment with denosumab were excluded (Figure 1). This left 51 patients for the present analysis: 22 male and 29 females. The median age was 37 years (range: 17–76 years) and the most frequent primary tumor locations were radius (12 cases) and sacrum + pelvis (14 cases) (Table 1 and Figure 2). All patients were treated with denosumab for longer than 1 year.

Eight/fifty-one (16%) patients had surgically unresectable GCTB (cohort 1) and underwent denosumab for a median of 65 months (range 23–85 months).

Forty-three/fifty-one (84%) had surgically resectable GCTB (cohort 2). Thirty-nine/forty-three (91%) patients with resectable disease underwent surgery after a median of 8 months (range 6 to 36 months): a resection in 7/39 and a curettage in 32/39. Six months post-operative treatment with denosumab was administrated as per the protocol.

### 3.2. Serum Bone Turnover Markers (sBMT)

**Figure 3:**
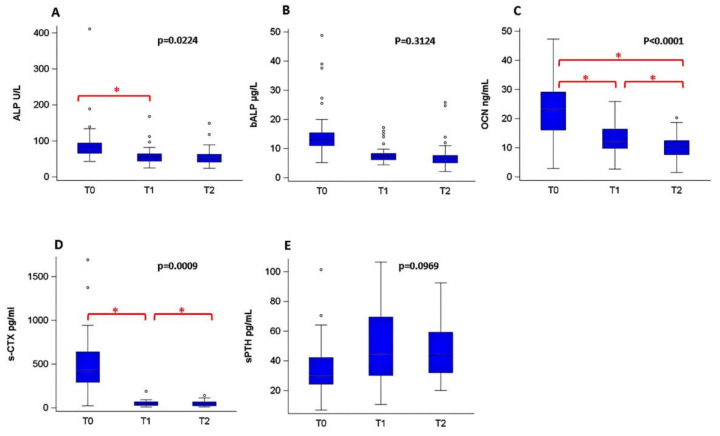
Serum bone turnover marker (sBTM) changes over time. Box-plot shows sBTMs measured at different time-points 0, 1, 2. Panel (**A**) for alkaline phosphatase (ALP), panel (**B**) for bone-alkaline phosphatase (bALP), panel (**C**) for osteocalcin (OCN), panel (**D**) for carboxyterminal-crosslinked-telopeptide of type I collagen (s-CTX) and panel (**E**) for parathyroid hormone (s-PTX). The line inside the box represents the median value, the height of the box is the interquartile range, and the circles represent the outlier values. The *p* value refers to the main effect of time from nonparametric repeated-measures analysis of variance (ANOVA). * Significant *p* value (*p* < 0.005) from post hoc multiple comparison test.

At baseline, the median level of BTMs were: s-CTX: 427 pg/mL (range 23–1692 pg/mL); ALP: 83 U/L (43–411 U/L); bALP: 13 μg/L; (5.2–48.8 μg/L); sPTH: 29 pg/mL (7–101.4 pg/mL); OCN 23 ng/mL (2.9–47.3 ng/mL) (Figure 3).

Higher median ALP and s-CTX at baseline were observed in patients with tumor size ≥ 5 cm as compared with patients with tumor size < 5 cm but the differences did not reach statistical significance (*p* = 0.0512 and *p* = 0.0589). (Table 2). No statistically significant difference was also found for sBMT median value at baseline for patients aged ≥50 years, versus those <50 years (ALP *p* = 0.67; bALP *p* = 0.72; sPTH *p* = 0.16; OCN *p* = 0.44; s-CTX *p* = 0.11). After denosumab ALP, OCN and s-CTX decreased at T1 and this reduction was confirmed at T2 for s-CTX and OCN. An increasing trend was observed for sPTH although not statistically significant (Figure 3).

### 3.3. Serum Bone Turnover Markers (sBMTs)

With a median follow-up of 59 months (IQR: 45–66), the 3-year DFS was 65% (95%CI 52–79), with a significantly worse outcome for patients with high (≥500 UI/mL) s-CTX at baseline, as compared to low s-CTX (<500 UI/mL) (3-year DFS for high s-CTX was 45% (95%CI 23–67) vs. 75% (95%CI 59–91) for low s-CTX). No differences were found in 3-year DFS according to bALP, ALP, OCN and sPTH (Table 3).

**Figure 4:**
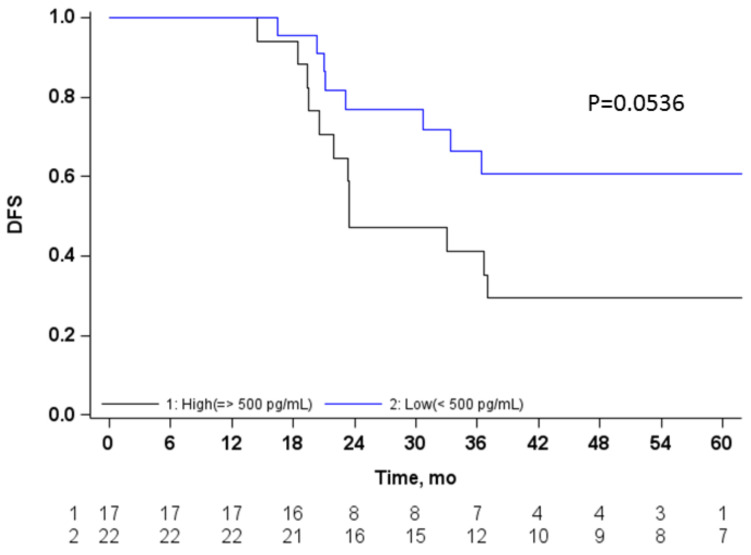
Disease-free survival (DFS) in 39 patients undergoing surgery for giant cell tumor of bone according to s-CTX at diagnosis.

In the subgroup of patients of cohort 2, at the time of analysis 20 out 39 (51%) were relapsed after surgery: 2/7, 29% after resection; and 18/32, 56% after curettage (*p* = 0.2351). The 3-year DFS was 35% (95%CI 15–57), for patients with high (≥500 UI/mL) s-CTX at baseline, and the 3-year DFS was 61% (95%CI 36–78) with low s-CTX (<500 UI/mL) (Figure 4).

## 4. Discussion

GCTB is an uncommon benign primary tumor that mainly affects long bone. It is locally aggressive and occasionally metastasizes to the lungs [16].

Surgery is the only potentially curative treatment option for patients with GCTB, but may be associated with severe morbidity and loss of function [17].

Increased RANKL in the bone microenvironment upregulates osteoclastogenesis and the activation of mature osteoclasts, resulting in increased bone resorption. The subsequent release of growth factors and calcium into the bone microenvironment stimulates additional proliferation of tumor cells and release of tumor-derived factors, and thus further increases the RANKL/OPG ratio, thereby promoting continued RANKL-dependent osteoclast-mediated bone destruction [18].

Denosumab, an human monoclonal antibody specific for RANKL, inhibits the formation, activation, and survival of osteoclasts, decreasing bone resorption and reducing cancer-induced bone destruction [19], and is approved for the treatment of patients with osteoporosis, bone metastases and GCTBs.

Furthermore, literature data suggest that genes involved in bone vascular formation, such as *OPG* and VEGF seem to have a potential role as new antiangiogenic generation drugs, including the multitarget tyrosine-kinase inhibitor lenvatinib. Interestingly, the combination of denosumab and lenvatinib seems to be a promising therapeutic strategy in GCTB [20,21].

This study demonstrated that s-CTX, ALP and osteocalcin OCN were significantly reduced after 3 months of treatment with denosumab in all patients with GCTBs.

The GCTB stromal cells are the major neoplastic and proliferative component of GCTBs and highly express receptor activation of nuclear factor-kappa β (RANK) ligand [22,23]. In bone osteolytic tumors, as well as GCTBs, the massive bone resorption is triggered by the RANKL/RANK axis that stimulates osteoclast-dependent and -independent pathways via activation of intracellular mediators [24].

The standard of care for GCTB is surgery (intralesional curettage or resection) with a high relapse rate [2,9]. The approval of denosumab has changed the therapeutic armamentarium for patients with GCTB [25]. While the role of denosumab in the setting of advanced and unresectable disease is well established, its role in surgically resectable disease is currently under discussion [26].

The ability to determine bone turnover with biochemical markers has been well known for several years [11] but data on BTM changes in patients with GCTBs undergoing denosumab treatment are lacking.

We aimed to describe BTM changes over time in GCTB patients undergoing treatment with denosumab within a phase II prospective clinical trial, in order to identify prognostic and early predictors of relapse.

Several studies analyzed the role of BMTs and tumor progression, in order to identify clinical validation prognostic biomarkers [8]. Serum bALP concentrations significantly correlated with the extent of bone involvement in bone metastases of several bone-homing tumors (breast, prostate and lung) [12]. Higher levels of serum bALP in patients with bone metastases as compared with patients without bone lesions have been shown [27]. Furthermore, high serum levels of bALP at baseline were associated with increased risks of disease progression in malignant carcinomas; in particular Lipton et al. [14] showed that after 3 months of treatment with denosumab, patients with bALP ≥ median value had a significantly reduced overall survival (OS) at 3 months, as compared with those who had bALP levels < median.

Bone resorption inhibitors, including denosumab, are associated with BMT reduction in several solid tumors. In a study on breast cancer, patients treated with denosumab had significant suppression of BMTs compared to baseline, and remained suppressed throughout the 2 years [28].

Interestingly, s-CTX high serum levels at baseline seems to represent a poor prognostic factor for patients undergoing surgery after neoadjuvant denosumab, with a 3-year DFS of 35% as compared to a 3-year DFS of 61% for those patients with low s-CTX. This result has a particular relevance due to the lack of association with increased s-CTX at baseline and size > 5 cm. This finding is in line with data on breast cancer, and Brown et al. demonstrated that high baseline s-CTX was prognostic for future bone recurrence at any time [29]. Baseline levels of s-CTX should be incorporated into GCTB patient work-up and might support clinical decisions such as the surgical approach. This finding is particularly relevant since the approval of denosumab has changed the therapeutic armamentarium for GCTB, but the optimal treatment strategy, including the role of neoadjuvant denosumab [26] and the type of surgery after denosumab-induced changes, is still to be defined. The treatment of choice for this subgroup of patients should be resection or aggressive curettage with adjuvant therapy, while simple curettage might be adequate for patients with low s-CTX at baseline [30]. Furthermore, these data might indicate that patients with an high baseline s-CTX would benefit from closer follow-up with CT or MRI as imaging of choice, within the first two years post-surgery, while patients with low-s-CTX at baseline might be followed with six-monthly plain radiograph X-ray. These suggestions should be confirmed in a larger series.

Denosumab lowered OCN in patients with GCTB, similar to what occurs in postmenopausal women treated with the same drug for osteoporosis [31]. Among BTMs, PTH showed an increasing trend by denosumab. Several factors secreted by tumor cells stimulate osteoclast activity and bone resorption and among them, PTH was the first to be recognized as involved in malignant osteolysis: in breast cancer cells, treatment with the antibody anti-PTH reduced the development of osteolytic lesions [32] by regulating the RANKL/RANK axis. In murine models, PTH improves bone mineral density by increasing osteoblast differentiation and reducing tumor cells migration [33]. In our series, PTH levels were increased after 3 months of denosumab treatment. Similarly, an increase in the PTH level has been reported in postmenopausal women undergoing denosumab for osteoporosis and is associated with greater bone mineral density and greater inhibition of bone remodeling compared with zoledronic acid [34]. Although not statistically significant, our findings revealed a positive correlation between high medium ALP at baseline and tumor size (>5 cm).

These factors are prognostic in another primary tumor [35]. The major limitation of this series is the lack of long-term BTM data, both for patients with inoperable GCTB undergoing continuous long-term denosumab treatment and for patients after denosumab interruption. Based on our findings, monitoring of BTMs after denosumab suspension and at relapse might offer some guidance on the treatment schedule, including monthly versus 3-monthly schedule or the discontinuation approach. This might be relevant since while denosumab was generally well tolerated, as reported [10], a dose-dependent increase in the risk of osteonecrosis of the jaw was demonstrated [10].

## 5. Conclusions

The use of denosumab in patients with GCTBs induces a generalized reduction in ALP, OCN and s-CTX. High baseline s-CTX, a marker of bone resorption, identifies a group of patients at higher risk of progression of the disease. These findings might guide identification of patients with higher risk of relapse and might support multidisciplinary team decisions on neoadjuvant use of denosumab.

## Figures and Tables

**Table 1:** Patient characteristics at baseline (*n* = 51).

Variation		Cohort 1 Unresectable (*n* = 8)	Cohort 2 Resectable (*n* = 43)
**Sex, *n* (%)**			
	Male	5 (62.5)	17 (39.5)
	Female	3 (37.5)	26 (60.5)
**Age**	Median	47.5	36
	Range	32–76	17–64
**Site, *n* (%)**			
	Femur	1 (12.5)	5 (12)
	Tibia	0	11 (26)
	Calcaneus	0	1 (2)
	Humerus	0	6 (14)
	Radius	0	12 (28)
	Ulna	0	1 (2)
	Pelvis Sacrum	7 (87.5)	7 (16)
**Size, *n* (%)**	≥5	6 (75)	30 (70)
	<5	2 (25)	13 (30)

**Table 2:** Baseline Bone Tumor Markers (BTMs) and tumor size (>5 cm). ALP (alkaline phosphatase); bALP (bone-alkaline phosphatase); s-PTH (parathyroid hormone); OCN (osteocalcin); s-CTX (carboxyterminal-crosslinked-telopeptide of type I collagen).

BTMs		Median (Min–Max)	*p* Value
ALP	<5 cm	68 (43–116)	0.0512
	≥5 cm	86.5 (53–411)	
			
bALP	<5 cm	12 (5.2–17.4)	0.3112
	≥5 cm	13 (8.3–48.8)	
			
s-PTH	<5 cm	35.7 (19.4–63.7)	0.1601
	≥5 cm	28.3 (6.9–101.4)	
			
OCN	<5 cm	20.4 (6–31.8)	0.6753
	>5 cm	23.4 (2.9–47.3)	
			
s-CTX	<5 cm	296 (23–943)	0.0589
	≥5 cm	502.5 (159–1692)	

**Table 3:** Disease-free survival (DFS) in patients with GCT of bone according to baseline serum levels of carboxyterminal-crosslinked-telopeptide of type I collagen (s-CTX), alkaline phosphatase (ALP), bone-alkaline phosphatase (bALP), parathyroid hormone (sPTH), and osteocalcin (OCN). * missing in 1 patient; ** missing in 3 patients.

Variable	N. Patients	% 3-Year DFS	95%CI	*p* Value
**Overall**	51	65	52–79	
s-CTX *				
High (≥500 pg/mL)	21	45	23–67	0.03
Low (<500 pg/mL)	29	75	59–91	
ALP *				
High (≥80 UI/L)	27	62	43–80	0.6
Low (<80 UI/L)	23	69	49–88	
bALP **				
High (≥12 μg/L)	27	69	51–87	0.5
Low (<12 μg/L)	21	65	44–86	
sPTH *				
High (≥30 pg/mL)	23	63	42–83	0.7
Low (<30 pg/mL)	27	66	48–84	
OCN *				
High (≥20 ng/mL)	29	65	48–83	0.9
Low (<20 ng/mL)	21	64	42–86	

## Data Availability

All data generated or analyzed during this study are included in this manuscript.
